# An Update on Drugs Used for Lumbosacral Epidural Anesthesia and Analgesia in Dogs

**DOI:** 10.3389/fvets.2017.00068

**Published:** 2017-05-12

**Authors:** Paulo V. M. Steagall, Bradley T. Simon, Francisco J. Teixeira Neto, Stelio P. L. Luna

**Affiliations:** ^1^Faculty of Veterinary Medicine, Department of Clinical Sciences, Université de Montréal, Saint-Hyacinthe, QC, Canada; ^2^Department of Small Animal Clinical Sciences, College of Veterinary Medicine and Biomedical Sciences, Texas A&M University, College Station, TX, USA; ^3^Faculty of Veterinary Medicine and Animal Science, Department of Veterinary Surgery and Anaesthesiology, Universidade Estadual Paulista (UNESP), Botucatu, Brazil

**Keywords:** epidural, canine, analgesia, anesthesia, opioids, local anesthetics, pain

## Abstract

This review aims to report an update on drugs administered into the epidural space for anesthesia and analgesia in dogs, describing their potential advantages and disadvantages in the clinical setting. Databases searched include Pubmed, Google scholar, and CAB abstracts. Benefits of administering local anesthetics, opioids, and alpha_2_ agonists into the epidural space include the use of lower doses of general anesthetics (anesthetic “sparing” effect), perioperative analgesia, and reduced side effects associated with systemic administration of drugs. However, the potential for cardiorespiratory compromise, neurotoxicity, and other adverse effects should be considered when using the epidural route of administration. When these variables are considered, the epidural technique is useful as a complementary method of anesthesia for preventive and postoperative analgesia and/or as part of a balanced anesthesia technique.

## Introduction

Epidural administration of drugs for pain management has been widely used in veterinary medicine (1–3). The advantage of this route is its proximity to the spinal cord receptors involved in the modulation and transmission of the nociceptive signal. The terms epidural and extradural refer to the space outside the dura mater. The terms intrathecal, subarachnoid, and spinal refer to the space between the pia mater and arachnoid membrane. *Epidural anesthesia* refers to the sensory, motor, and autonomic blockade produced by epidural administration of local anesthetics while *epidural analgesia* refers to epidural administration of analgesics, such as opioids.

The use of this technique may provide preemptive analgesia by inhibiting central sensitization and modulating afferent signals to the dorsal horn, reducing pain and inhalant, and/or analgesic requirements during the perioperative period (4–6). In addition, epidural anesthesia suppresses the markers of stress response as represented by decreases in serum concentrations of cortisol and norepinephrine for up to 48 h after administration (7–9).

Absolute contraindications to epidural anesthesia include untreated hypovolemia, coagulation disorders, septicemia, bacteremia, skin trauma, neoplasia, and/or infection at the lumbosacral region (2, 10). Some neurological diseases, spinal cord trauma, low-dose heparin therapy, and anatomical changes including trauma of the pelvic area and obesity resulting in difficulty locating the lumbosacral space are considered relative contraindications (2). The use of ultrasonography to locate the epidural space may circumvent this latter issue (11).

The benefits of this technique often outweigh the risks; however, clinicians should be aware of potential complications such as technical failure, contamination, hematoma, accidental intrathecal injections, respiratory depression, sympathetic blockade, bradycardia, hypotension, neurological deficits, delayed hair growth, pruritus, and urinary retention (12–18). For example, an epidural abscess and lumbosacral discospondylitis has been reported in a dog most likely due to colonic perforation or fecal contamination (19). The technique was performed after multiple skin penetrations and subcutaneous needle redirections were done prior to achieving correct epidural placement. Epidural injections should only be performed after strict aseptic technique and manipulations should be gentle and cautious. In addition, epidural injections should be performed with preservative-free solutions to avoid the toxic effects produced by preservatives on the spinal cord. However, in research settings, epidural administration of morphine containing 0.1% of sodium metabisulfite did not cause neurotoxicity in dogs (20). In humans, side effects after intrathecal administration of morphine containing up to 0.004% sodium edetate and up to 0.04% of sodium metabisulfate appeared to be non-existent in 15 patients (21). In rats and sheep, chronic intrathecal administration of neostigmine containing methyl- and propylparaben did not produce any histologic evidence of neurotoxicity. Some institutions in North America and Europe use morphine containing 0.1% of sodium metabisulfite by the epidural route on a routine basis in dogs without reporting side effects (9).

Epidural administration of local anesthetics can be effective for a variety of surgical procedures such as cesarean section (22), orthopedic procedures in the hind limb, and soft tissue surgeries (8, 23). In these cases, minimal maternal and fetal depression (22) and less cardiopulmonary depression than general anesthesia (23) have been observed. Epidural anesthesia has been frequently used in combination with general anesthesia to reduce anesthetic and analgesic requirements, improve analgesia, and quality of anesthetic recovery (8, 24–26).

This review reports potential advantages and disadvantages of each class of drugs and their combinations used for epidural anesthesia and analgesia in dogs. The epidural technique *per se* is not the main focus of this article and has been reported elsewhere (10, 27).

## Local Anesthetics

Local anesthetics diffuse across the dura mater to act on nerve roots and the spinal cord, and into the paravertebral area through the intervertebral foramina, producing multiple paravertebral nerve blocks after epidural administration (28).

### Factors Influencing Onset and Duration of Autonomic, Sensory, and Motor Blockade

The extent of the sensory, motor, and autonomic blockade produced by a lumbosacral epidural injection will depend on the cranial distribution of the local anesthetic according to the anesthetic volume, concentration, velocity of injection, amount of the epidural fat, epidural space size, posture, and gravity (29–35). For example, the use of low concentration of local anesthetics (e.g., bupivacaine less than 0.25%) or low-dose bupivacaine (0.05 mL kg^−1^) may be useful when sensory blockade is desired with minimum impairment of motor function (36–38). This property of “differential” or “selective” blockade may be attributed to the larger diameter and more profound myelination of Aα motor nerve fibers when compared with Aδ and C fibers (39). However, this is not always the case and motor function may be blocked even when low concentrations are administered (40).

It has been recommended that dogs be positioned in sternal recumbency and kept in this position following injection to provide bilateral analgesia or in lateral recumbency with the surgical site as the dependent side until the block has taken effect (32). One study using canine cadavers showed that if greater cranial migration of an epidurally administered drug is desired, placing the patient in lateral recumbency with the surgical site on the dependent side for 30–40 min may precede surgery (gravity effect) (32). In the authors’ experience, placing the affected limb in a dependent position for the duration of the clipping and surgical preparation (approximately 30 min) is enough time to achieve this effect without delaying surgery.

When local anesthetics are administered epidurally using a Tuohy needle for epidural catheter placement, the needle bevel should face the head of the animal since it will facilitate the threading of the catheter to more cranial portions of the spinal canal (41). However, there is a lack of evidence to support the benefit of this technique when an epidural catheter is not being used, as the Tuohy needle bevel direction had limited clinical significance on preferential spread of 1.5% lidocaine after epidural administration in young (<40 years of age) human subjects (42). Another factor that may play a role in the cephalad migration of the local anesthetic in the spinal canal is the velocity of injection. However, a recent study showed that an injection velocity of 1 mL min^−1^ compared with 2 mL min^−1^ of 0.5% bupivacaine after epidural administration had limited effect on injectate distribution and sensory blockade in anesthetized dogs (33). The effect of epidural drug injection velocity on duration and quality of analgesia is yet to be determined (43). In the authors’ routine, injectate volume is commonly administered over approximately 1 min. Peak cephalad migration of the injectate to the level of the cervical vertebra within the spinal canal is typically reached at doses of 0.4 mL kg^−1^ in dogs (40). Patients with high body mass index may have a smaller volume of epidural space due to accumulation of adipose tissue in the spinal canal (34, 44). This factor may contribute to a more cranial migration of the local anesthetic specifically with injections in the lumbosacral region (45). Lean body weight or spinal column length (occipital condyle to the first coccygeal vertebra) should be used when calculating the epidural dose in obese patients or those with abnormal spinal column conformations (35). An upward sloping body position of patients in sternal recumbency also did not affect injectate spread (44).

### Side Effects after Epidural Administration of Local Anesthetics and Their Treatment

In general, small volumes (0.1 mL kg^−1^) of 2% lidocaine or 0.5% bupivacaine produce blockade of the immediate lumbosacral area while larger volumes (0.2 mL kg^−1^) produce complete desensitization of the caudal portions of the abdominal cavity, inguinal area, hind limbs, tail, and perineum (30, 46). Although volumes of local anesthetic exceeding 0.2–0.25 mL kg^−1^ may desensitize areas up to the thoracic and cervical vertebrae (31, 40), the use of greater volumes is not recommended because long-term blockade of sympathetic fibers may lead to severe cardiovascular depression, characterized by peripheral vasodilation, hypotension and bradycardia (47), and respiratory compromise as a result of blockade of the phrenic nerve (48). Hypovolemia should be treated before epidural anesthesia and cardiorespiratory monitoring is strongly recommended during injection otherwise serious complications may occur. Cardiac arrest due to excessive sympathetic blockade has been reported in a dog with uncorrected hypovolemia after lumbosacral administration of epidural 2% lidocaine (5.33 mg kg^−1^) (49). Extensive cranial spread of epidural administration of 0.75% ropivacaine resulting in high sympathetic blockade (levels T1–T3) was likely to be the cause of cardiovascular depression and the Horner’s syndrome in a dog (13). Horner’s syndrome, ataxia, paraplegia, depression, stupor, and intermittent cough were also reported in dogs receiving epidural lidocaine (0.1–0.2 mL kg^−1^) at the level of the seventh *thoracic* epidural space (50). The thoracic epidural space for the administration of anesthetics or analgesics has received little attention in small animal medicine, but may provide an alternative to the lumbosacral approach when analgesia for the thoracic or upper-abdominal region is required (50, 51). To avoid cephalad spread and adverse effects associated with the thoracic epidural technique, smaller volumes (0.1 mL kg^−1^) should be considered when compared to the lumbosacral approach. In the authors’ experience, complications are rare with lumbosacral epidural administration and avoided by following appropriate dosage regimens (Table 1) and technique.

**Table 1:** **Commonly used and recommended epidural anesthetics and analgesics in dogs**.

Drug	Dose (mg kg^−1^)	Final volume (mL kg^−1^) injected into the LS epidural space	Onset time (min)	Duration of analgesia (h)	Comments	Reference
**Local anesthetics**						
2% Lidocaine with 1:200,000 epinephrine	5.0	0.25	4–6	1	Duration of motor blockade 60–120 min	(46, 60, 66, 68)
0.5% Bupivacaine	0.5–1.0	0.2–0.25	5–15	>2	Duration of complete motor blockade and ataxia was 65 and 240 min, respectively. May be prolonged with 0.75% bupivacaine. Complete motor blockade may not be observed at 0.25%	
0.5% Levobupivacaine	0.5–1.0	0.2	5–15	1–1.5	Duration of complete motor blockade and ataxia was 30 and 180 min, respectively. Complete motor blockade may not be observed at 0.25%	
0.75% Ropivacaine	1.65	0.22	7–15	1.5–2.5	Duration of motor blockade 90–150 min	
**Opioids**						
Morphine PF	0.1	0.1 for abdominal and pelvic procedures; 0.25 for thoracic procedures	45–90	12–24 for pelvic limb and abdominal procedures at 0.1 mL kg^−1^; 5–6 for thoractomy procedures at 0.25 mL kg^−1^	Reduced minimum alveolar concentration (MAC) by 30% and minimized CV depression from inhalant. Potential for urinary retention and pruritus	(4, 25, 80)
Buprenorphine	0.004	0.2	<60	Up to or greater than 24	Reduced risk for urinary retention	(83)
**Local anesthetics and opioids**						
Morphine PF and 0.5% bupivacaine	0.1 and 0.5–1.0	0.22	<15	Up to 24	67% return to normal motor function within 8 h. Potential for urinary retention	(125, 126)
Oxymorphone and 0.75% bupivacaine	0.1 and 1.0	0.2	<15	Up to 24	Decreases in heart rate. Transient hypotension. Systemic absorption of epidural oxymorphone is high	(24, 88)
Buprenorphine and 0.5% bupivacaine	0.004 and 1.0	0.2	<30	Up to 24	Low incidence of urinary retention	(130)
**Alpha_2_-Adrenoreceptor agonists**						
Dexmedetomidine	0.003–0.006	0.25	<15	Up to 4.5	Dose-dependent MAC reduction up to 4.5 h. Bradycardia and elevated blood pressure may occur. Minimal effects on motor function	(110, 111)
**Alpha_2_-Adrenoreceptor agonists and local anesthetics**						
Dexmedetomidine and 0.5% bupivacaine	0.004 and 1.0	0.22	<15	Up to 24	Less urinary retention when compared to opioid epidurals	Prolonged motor blockade compared to local anesthetic and opioid epidurals	(126)

The prevalence of cardiovascular adverse effects has been reported in the literature (6). For example, mild cardiovascular depression developed in 20 of 219 dogs receiving a lumbosacral epidural injection of bupivacaine and morphine during inhalation anesthesia (6). Morphine does not cause sympathetic blockade and therefore it is likely that the cardiovascular depression reported in that study was associated with the blockade of sympathetic ganglia induced by the local anesthetic. Epidural administration of a large volume of 0.75% ropivacaine (1.65 mg kg^−1^ or 0.22 mL kg^−1^) at the *thoracic* level was also shown to produce hypotension and bradycardia in isoflurane-anesthetized dogs (17, 52). Transient hypotension and bradycardia have also been reported in two obese dogs after lumbosacral epidural administration of local anesthetics (16). Although the volume injected into the epidural space was 0.2 mL kg^−1^ in both dogs, the total volume administered was relatively high (8 and 11 mL) (16). Cardiovascular depression may be observed due to the spread of local anesthetic to the upper four thoracic segments, low right-sided cardiac filling pressure, high epidural pressure raising cerebrospinal fluid (CSF) pressure resulting in increased vasomotor tone, and due to undetected hypovolemia and/or rapid and high volume injection of drugs into the epidural space (16). There is usually a recommendation by veterinarians without scientific support of limiting total injectate volume of local anesthetics to a maximum of 6 mL in dogs weighing more than 30 kg (10). However, larger volumes have been reported (8.6 mL) without complications (53). The authors have also used higher volumes (e.g., 8 mL) of epidural injectate in large canine breeds without complications but one should consider that smaller volume per kg of bodyweight (less than 0.2 mL kg^−1^) is often enough for pelvic limb surgery.

Post-epidural hypotension during combined epidural/general anesthesia (systolic arterial blood pressure < 90 mmHg or mean arterial pressure < 60 mmHg) might be treated by increasing intravenous crystalloid administration and reducing vaporizer concentrations. In humans, post-epidural hypotension was found to be very frequent (55–90%) in patients undergoing cesarean section and spinal anesthesia (54). A crystalloid or colloid preload alone was ineffective in preventing hypotension following the administration of the local anesthetic (17, 54). Ephedrine (0.05 mg kg^−1^ IV), a non-selective agonist of beta and alpha receptors increases mean arterial blood pressure due to increases in systemic vascular resistance and cardiac output, may be effective in the treatment of post-epidural hypotension. Ephedrine has the advantages of being inexpensive and convenient to use as an intravenous (IV) bolus. Given the transient nature of hypotension observed after epidural anesthesia, the authors have found that a single dose is adequate in most cases. Ephedrine significantly increased cardiac index and stroke volume index but not arterial blood pressure in 5 out 6 dogs that had been treated with an epidural with morphine–bupivacaine (55) but one has to consider that this was a small clinical study where different anesthetic protocols had been administered. If ephedrine is ineffective to treat post-epidural hypotension, dopamine can be administered as a constant rate infusion (5–10 μg kg min^−1^) as the rate can be adjusted according to individual blood pressure responses. If bradycardia-induced hypotension is detected, a reflex as a result of either low right heart filling pressures or from the generation of high extradural pressures following an epidural, it can be treated with the administration of an anticholinergic such as atropine (0.02–0.04 mg kg^−1^ IV).

### Drugs

Table 1 shows the local anesthetics used for epidural anesthesia in dogs.

Lidocaine is an amide type local anesthetic that can be administered into the lumbosacral epidural space to produce a rapid desensitization with good muscle relaxation (46). Epidural administration of 0.25 mL kg^−1^ of 2% lidocaine with epinephrine (1:200,000) results in shorter latency period and shorter duration of desensitization, than 0.25 mL kg^−1^ of 0.5% bupivacaine with epinephrine (1:200,000) (46). Because the duration of sensory blockade is longer following epidural bupivacaine 0.5% alone (110 ± 14 min) or bupivacaine 0.5%–lidocaine 2% (94 ± 8 min) when compared with lidocaine alone (54 ± 5 min), the use of epidural bupivacaine either alone or combined with lidocaine may be preferred during prolonged surgeries (46). Onset of analgesia has been shown to be clinically similar following epidural administration of 2% lidocaine alone (5 ± 1 min) or in combination with 0.5% bupivacaine (7 ± 1 min) (46).

Bupivacaine is another amide type local anesthetic with a prolonged duration of effect compared with lidocaine (56) and may induce ventricular arrhythmias and cardiotoxicity at lower doses than lidocaine (57) but this is usually not a concern when doses and concentrations stated in this review are followed. At clinically used doses, epidural bupivacaine has limited effects on ventilation and oxygenation (24). Delayed hair growth following epidural bupivacaine has been reported in dogs (6, 14). However, this is observed following any epidural injection and not only with bupivacaine. Other factors such as season, housing, diet, or care of the coat may contribute to delayed hair growth following lumbosacral epidurals in dogs (14). The epidural administration of 0.2 mL kg^−1^ or 0.22 mL kg^−1^ of 0.25% or 0.75% bupivacaine results in analgesia to the level of the third or fourth lumbar vertebra, respectively (40, 58). A larger volume (0.31 mL kg^−1^) is required to anesthetize up to the eleventh thoracic vertebra (59) but this volume may not be recommended for clinical use because sympathetic blockade followed by hypotension may result especially during the combine used of epidural anesthesia with inhalational anesthetics. The onset of motor blockade after lumbosacral administration of 0.5% bupivacaine (1.8 mg kg^−1^) is approximately 2–4 min and lasts about 2–3 h (60).

Ropivacaine is a highly lipid soluble local anesthetic with the advantage of being less arrhythmogenic and toxic for the CNS and cardiovascular system than bupivacaine. Of interest, both drugs have similar pharmacokinetic profiles (61–64). It is believed that ropivacaine causes less motor blockade than bupivacaine in humans (65) and dogs (29, 66), but this has not been confirmed in clinical trials using dogs. To obtain a successful blockade (>80%) up to dermatomes T13–L1, a volume of 0.22 mL kg^−1^ using the concentration of 0.75% of bupivacaine or ropivacaine has been recommended (30).

Levobupivacaine is the S (-) isomer of bupivacaine and produced longer duration of motor block when compared with racemic bupivacaine in dogs (67). However, the dose-dependent analgesic and motor-blocking effects of epidurally administered levobupivacaine and racemic bupivacaine (0.25, 0.5, and 0.75%) were similar in conscious dogs (68). Like ropivacaine, levobupivacaine is less arrhythmogenic and cardiotoxic than bupivacaine (63).

### Adjuvants Drugs without Intrinsic Analgesic Effect in Combination with Local Anesthetics

The duration and intensity of desensitization produced by the epidural technique will be influenced by the addition of adjuvant drugs without intrinsic analgesic effect. Epinephrine (1:200,000 or 5 μg mL^−1^) aims to increase the duration of action and reduce the incidence of adverse effects by reducing systemic absorption of the local anesthetic from the injection site (69–71). In dogs, the epidural administration of mepivacaine with epinephrine (1:200,000) exerted considerable influence on the azygos venous blood uptake: peak concentrations of mepivacaine were reduced by two-thirds and systemic absorption of the drug was delayed. The azygos vein collects most of the blood draining from the internal vertebral venous plexus; therefore, the concentration of local anesthetics in this vein is an approximation of the total uptake by the epidural plexus (69). However, this effect may not be seen when epinephrine is added to long acting local anesthetics such as bupivacaine and ropivacaine, presumably because the greater solubility of these drugs causes them to bind avidly to tissues. In one study, the sensory and motor blockade produced by epidural administration of 0.75% bupivacaine or 1% ropivacaine in dogs was not further prolonged when epinephrine (1:200,000) was added to the local anesthetic solutions. However, epinephrine reduced the average anesthetic blood concentration observed in both treatment groups (62) possibly decreasing the likelihood of systemic toxicity. In a more recent study, dogs undergoing tibial plateau leveling osteotomy that received an epidural combination of ropivacaine, sufentanil, and epinephrine had longer lasting analgesia with less adverse effects when compared to epidural ropivacaine alone or combined with sufentanil (71). Therefore, the addition of epinephrine (1:200,000) to local anesthetics is an alternative to prolong duration of action of standard formulations of local anesthetics. However, in some cases, this may be a disadvantage especially if motor tone and normal ambulation are impaired hours after surgery.

## Opioids

Opioids act by binding to opioid receptors that are located in the central nervous system, spinal cord, and peripheral tissues. Epidural or spinal administration of opioids relieve somatic and visceral pain by selectively blocking nociceptive impulses without interfering with sensory and motor function or depressing the sympathetic nervous system (72). The pharmacodynamic profile is based on their receptor specificity, affinity, potency, and efficacy at the different opioid receptors and has been discussed elsewhere (73, 74).

### Drugs

Highly hydrophilic drugs, such as morphine, produce analgesia 30–40 min after epidural administration and have long duration of effect due to the delayed systemic absorption and longer maintenance in the spinal cord (75, 76). Highly lipophilic drugs, such as fentanyl and meperidine have a shorter latency and duration of action (73, 77, 78). Lipophilicity can increase uptake into the adipose tissue of the epidural space, the degree of which will depend on the quantity of fat around the epidural space. Therefore, the tendency of drugs with high lipid partition coefficients to bind into the lipid phase leads to drug sequestration in lipid layers.

In dogs, epidural administration of 0.1 mg kg^−1^ of morphine reduced the minimum alveolar concentration (MAC) of halothane by 30% and minimized the hemodynamic depression produced by the inhalant agent (4, 79). Since morphine is more soluble in water than in fat, epidural administration of this opioid results in relatively high concentrations of the drug in the CSF (76). The tendency for epidural morphine to accumulate in the CSF rather than in fat results in delayed systemic absorption and facilitates the cranial spread of this opioid in the vertebral canal (76). These unique pharmacokinetic properties of epidural morphine (long residence time in the CSF and delayed systemic absorption) explain why lower doses of this opioid administered epidurally (0.1 mg kg^−1^) will often result in more prolonged analgesic effects than those observed after systemic administration of higher doses and the extension of the analgesic effects to more cranial dermatomes, including the chest wall and thoracic limbs (75, 76, 80). In one study, epidural morphine (0.1 mg kg^−1^) produced similar analgesia when compared with intercostal bupivacaine during the first 24 h after thoracotomy (75) and both techniques are often combined by the authors for this type of surgery. Epidural administration of morphine may not be used as a single analgesic technique to treat severe postoperative pain. In the two studies comparing the postoperative analgesic effects produced by epidural morphine (0.1–0.2 mg kg^−1^) in combination with bupivacaine with those produced by epidural morphine alone (0.1–0.2 mg kg^−1^) or epidural saline (placebo), the opioid-local anesthetic combination was always associated with longer lasting analgesia and improved pain scores than the other epidural treatments (8, 25). Epidural administration of morphine also provides analgesia for castration procedures (81). However, the authors do not recommend this technique for routine neutering (castration or ovariohysterectomy) since pain after neutering is well controlled with the administration of nonsteroidal anti-inflammatory drugs, parenteral opioids, and other local anesthetic blocks (intratesticular or intraperitoneal blocks).

Buprenorphine is a promising drug for epidural analgesia (82) with similar analgesic effect and duration of action when compared to morphine in dogs undergoing stifle surgery (83). Epidural buprenorphine may be useful for some animals because it does not appear to induce the urinary retention due to detrusor muscle relaxation seen with epidural morphine (84). It is apparent that in dogs and humans (83, 85), epidural buprenorphine may induce long lasting analgesia of similar duration of that provided by epidural morphine, but with fewer side effects. However, additional studies are necessary to confirm if the epidural administration of this opioid can replace epidural morphine in dogs.

Other opioids including different formulations (e.g., methadone, liposome-based sustained-release preparations of morphine, oxymorphone, butorphanol, and tramadol) have been administered by the epidural route in the experimental and clinical setting but do not provide consistent and additional information that would replace or change current practices (86–92).

### Spinal (Intrathecal) versus Epidural Administration of Opioids

Spinal administration of opioids is not commonly used in small animals; however, it may provide some advantages over the epidural route such as lower intraoperative analgesic failure and faster return to normal motor function (93). A case report described the combination of spinal administration of fentanyl (0.77 μg kg^−1^) and bupivacaine (0.31 mg kg^−1^) and epidural administration of morphine (0.15 mg kg^−1^) for tail resection and surgical exploration of the pelvic canal in a dog (94). In addition, a sparing effect of a low dose of spinal morphine (0.03 mg kg^−1^) on intraoperative fentanyl requirements in dogs undergoing spinal surgery has been reported (95) but one should consider that dogs in the control group were intentionally given a higher loading dose and constant rate infusion of fentanyl when compared with the morphine group. The observer was also aware of the treatments administered (95). In both studies (94, 95), the authors reported good intra- and postoperative analgesia without neurological complications. However, spinal administration of opioids may not provide any clinical advantages over a single epidural injection of bupivacaine and/or morphine in most of the cases.

The anatomical location in which the opioid has been administered may also affect duration of analgesia. In one study, 0.1 mg kg^−1^ of morphine administered at T5–T6 provided significantly longer analgesia (9.9 ± 1.6 h) with minimal side effects when compared with its administration at L6–L7 (5.8 ± 0.8 h) following thoracotomy (80). This difference in duration of action may be attributed to its “local” effect when administered adjacent to the site of pain modulation. The use of topical epidural morphine administered at the site of injury *via* gelfoam, gelatin sponge, or “splash” technique resulted in varying effects on analgesia during hemilaminectomy in dogs (96–98). The administration of morphine (0.1 mg kg^−1^) or oxymorphone (0.1 mg kg^−1^) on a gelfoam pad then placed topically in the epidural space at the surgical site is used at the authors’ institutions in dogs undergoing hemilaminectomy as part of a multimodal analgesic protocol.

### Side Effects and Their Treatment

Side effects are not common but may occur following administration of opioids by the epidural route. Mild respiratory depression has been reported after epidural administration of 0.1 mg kg^−1^ of morphine in dogs (6) but this is not clinically relevant (38) if assisted or mechanical ventilation is promptly instituted. Pruritus has also been reported following epidural and intrathecal morphine administration (99–101). The use of a 5-hydroxytryptamine antagonists (e.g., ondansetron) has been described as treatment for neuraxial opioid-induced pruritus (102). Myoclonic activity upon recovery from anesthesia was also reported following intrathecal administration of morphine in dogs (15, 100) but this is not a clinical issue with epidural injections, providing that the maximum dose of 0.1 mg kg^−1^ is respected and accidental puncture of the subarachnoid space does not take place during needle insertion (103). Studies showed that approximately 3% of dogs receiving epidural morphine showed urinary retention postoperatively (6, 104). The prevalence of urinary retention increases when morphine is administered epidurally for postoperative pain control *via* indwelling epidural catheter. In this case, abnormal urinary patterns were observed in up to 39% of canine individuals (105). In practice, gentle bladder expression after surgery and before patient extubation avoids atony of the detrusor muscle and urinary retention (15).

Epidural administration of opioids seems to be a valuable tool for treatment of postoperative pain mainly as part of a balanced analgesia protocol and not as a sole technique. The dose, time to effect, and duration of action of epidural administration of commonly used opioids are described in Table 1.

## Agonists of Alpha_2_-Adrenergic Receptors

These drugs are potent sedatives and analgesics that bind to noradrenergic receptors of the spinal cord, inhibiting the central transmission of the afferent nociceptive impulses, by pre- and postsynaptic membrane hyperpolarization and inhibition of norepinephrine and substance P release (5, 106). The use of lower doses of epidural alpha_2_ agonists minimizes the systemic side effects and produces a more effective antinociception when compared with their systemic use (5, 106).

### Drugs

The MAC of isoflurane was reduced by 8, 22, and 33%, after epidural administration of 0.1, 0.2, and 0.4 mg kg^−1^ of xylazine, respectively (107). All doses of xylazine decreased the heart rate, but only the highest dose caused second-degree atrioventricular block (107). Epidural xylazine was also shown to provide longer postoperative analgesia when compared with epidural romifidine, dexmedetomidine, detomidine, and clonidine in dogs (108). This can be attributed to xylazine’s local-like anesthetic properties and its low lipophilicity when compared with other alpha_2_ agonists.

Medetomidine might produce dose-dependent antinociception when administered by the epidural route. Postoperative analgesia was observed for up to 90 min after epidural administration of 10 μg kg^−1^ of medetomidine in dogs (109) whereas 5 μg kg^−1^ of the drug administered by the epidural route did not provide analgesia when a towel clamp was applied to the tail (106). Epidural administration of medetomidine (15 μg kg^−1^) also caused bradycardia, atrioventricular block, and transient hypertension (5). Epidural dexmedetomidine (1 μg kg^−1^), the active isomer of medetomidine with a high alpha_2_-selectivity and efficacy, produced an antinociceptive effect and reduced heart rate, without changes in carbon dioxide response, behavior, and motor function (110). Epidural administration of dexmedetomidine to isoflurane-anesthetized dogs decreased the MAC of the inhalant anesthetic in a dose-dependent fashion (111).

Epidural administration of these drugs will normally cause mild to moderate cardiovascular changes which may or may not be tolerated depending on the health status of the patient (112). In addition, there are concerns of using multi-vial drugs (risk of contamination and neurotoxicity) with preservatives for epidural administration. A study showed that epidural administration of preservative-free dexmedetomidine to rabbits may produce spinal neurotoxicity, characterized by demyelination of the oligodendrocytes in the white matter (113). The neurotoxic effect was attributed to vasoconstriction of the medullary spinal vessels and pH of dexmedetomidine that can cause demyelination (113). The prolonged duration of block may be also an issue after administration of these drugs in combination with local anesthetics (see below). The dose, time to effect, and duration of action of epidural administration of commonly used alpha_2_-adrenoreceptor agonists are described in Table 1.

## Dissociative Anesthetics

Ketamine is a non-competitive antagonist of N-methyl D-aspartate receptors involved in the transmission and modulation of nociceptive stimuli in the dorsal horn of the spinal cord, which plays a key role in chronic pain, reducing spinal cord hyperexcitability (114–116). Ketamine has intrinsic local anesthetic effects, producing motor, sensory, and sympathetic block when administered by the epidural route (115). Epidural administration of ketamine (2 mg kg^−1^) caused minimal hemodynamic effects in dogs (114) and effectively reduced post-incisional hyperalgesia in the hind limbs for 12 h in dogs (117), but was more effective when administered preemptively (118). The advantages of epidural administration of ketamine over the systemic route in human medicine are controversial (119). Also, the efficacy of epidural racemic or ketamine S (+) has rarely been reported in veterinary medicine (117, 118). Preservative-free ketamine (S+) resulted in toxic damage to both the spinal cord and nerve roots in both rabbits and humans following repeated intrathecal administration (120, 121). Indeed, ketamine should not be administered by the epidural or intrathecal route until further studies report its neurotoxic effects in dogs.

## Opioid and Local Anesthetic Combinations

The combination of opioids with local anesthetics may be beneficial because affinity of opioid drugs for their receptors in the spinal cord is increased by local anesthetics (122). The combination of opioids and local anesthetics decreased anesthetic requirements, postoperative analgesic requirements, and plasma cortisol concentrations and improved pain scores in dogs undergoing surgery when compared with dogs that received an opioid alone (6, 8, 24, 25, 123, 124). The combination morphine–bupivacaine provides similar analgesia to femoral-sciatic nerve blocks or constant rate infusions of analgesics in dogs undergoing orthopedic surgeries (125–128). However, the drug combination may result in more intraoperative hypotension and urinary retention than other forms of local regional techniques due to the effects of local anesthetics and morphine on vascular and bladder tone, respectively (125). Epidural administration of morphine (0.1 mg kg^−1^) and 2% lidocaine (0.1 mL kg^−1^) may be an option when shorter duration of motor blockade is desired (129). If urinary retention is a concern, the administration of epidural 0.5% bupivacaine (1 mg kg^−1^) and buprenorphine (0.004 mg kg^−1^) provided up to 24 h of analgesia in dogs undergoing stifle arthroplasty with a low incidence (1 out of 13) of urinary retention (130).

Other combinations using local anesthetics and different opioids have been reported (e.g., methadone–lidocaine, morphine–ropivacaine, tramadol–lidocaine, oxymorphone–bupivacaine) but it seems that none of these protocols provided clear benefits over the administration of morphine–bupivacaine (64, 88, 127, 131, 132). However, they may be useful when the latter two drugs are not available.

The administration of local anesthetics and opioids can be also performed *via* a constant rate infusion using an epidural catheter. In this case, bupivacaine should be diluted to a concentration less than or equal to 0.125% and will avoid motor weakness than when higher concentrations are used. When combined in a 50:50 mixture with morphine (1 mg mL^−1^), 0.25% bupivacaine is diluted to 0.125% and when administered *via* an indwelling epidural catheter to deliver 0.3 mg kg^−1^ per day of morphine, the total amount of bupivacaine delivered to the patient is 0.75 mg kg^−1^ per day (12).

## Alpha_2_-Adrenoreceptor Agonist and Opioid or Local Anesthetic Combinations

The combination of alpha_2_-adrenoreceptor agonists and opioids into the epidural space increases magnitude of analgesia. This synergism may be due to the activation of alpha_2_-adrenoreceptors on opiate-containing interneurons, increasing the release of opioid peptides (133) and also due to the prolongation of the increased plasma morphine concentration produced by medetomidine (134). The duration of morphine analgesia was increased by almost twofold when morphine was administered with medetomidine in dogs (106). According to the authors, this may have been due to the waning alpha_2_-adrenoreceptor-mediated vasoconstriction associated with medetomidine in epidural vasculature. Morphine present in the spinal cord, CSF, and epidural tissues may have had increased uptake into systemic circulation following the relative vasodilation of epidural vessels as the medetomidine effect subsided (134).

The addition of 0.05% dexmedetomidine (4 μg kg^−1^) to 0.5% bupivacaine (1 mg kg^−1^) provided adequate analgesia; however, prolonged duration of motor blockade was a clinical concern following pelvic orthopedic surgeries in dogs (126). Time to first urination was earlier but pain scores worsened at 1 h following surgery when compared with dogs receiving epidural bupivacaine–morphine for similar procedures (126). The combination of alpha_2_ agonists with opioids or local anesthetics might be a good option for pain management in healthy dogs that are in severe pain. However, the same concerns described with the administration of agonists of alpha_2_ receptors alone should be taken in consideration.

## Dissociative Anesthetic and Opioid or Local Anesthetic Combinations

Ketamine might have an analgesic sparing effect when combined with local anesthetics and opioids (115). However, there was no potential advantage when opioids and ketamine were combined in humans (116) neither when ketamine (2.5 mg kg^−1^) was administered with meperidine (2 mg kg^−1^) by the epidural route of administration in dogs (135). On the other hand, levorotatory ketamine (ketamine S+) (0.5 or 1 mg kg^−1^) combined with morphine (0.025 or 0.05 mg kg^−1^) provided satisfactory intra- and postoperative analgesia with cardiorespiratory stability in dogs undergoing ovariohysterectomy (136). However, pain associated with ovariohysterectomy is moderate and the advantages of the use of epidural ketamine alone or combined with other drugs should be an objective of further studies.

Doses, injection volumes, onset period, and duration of epidural anesthesia or analgesia produced by combinations of drugs are described in Table 1.

Other drugs such as midazolam, ketorolac, and neostigmine have been administered epidurally in dogs in the experimental and clinical setting (137–141). None of these drugs have been shown to be promising safe and alternative analgesic techniques for clinical use.

## Conclusion

The potential for cardiorespiratory compromise, neurotoxicity, and other side effects should be considered when drugs are to be administered into the epidural space. These drugs should be selected according to the potency, onset and duration of action, surgical procedure, cardiorespiratory changes, and local and systemic side effects. When these variables are considered, the epidural route is useful as a complementary method of anesthesia, preemptive and postoperative analgesia, and/or as part of a balanced anesthesia technique.

## Author Contributions

PS, BS, FN, and SL have contributed to gathering data and writing of the manuscript; have thoroughly evaluated the final product and checked all references; PS and BS assisted with the manuscript publication process.

## Conflict of Interest Statement

The authors declare that the research was conducted in the absence of any commercial or financial relationships that could be construed as a potential conflict of interest.
